# Photophysical Behaviors of Single Fluorophores Localized on Zinc Oxide Nanostructures

**DOI:** 10.3390/ijms130912100

**Published:** 2012-09-24

**Authors:** Yi Fu, Jian Zhang, Joseph R. Lakowicz

**Affiliations:** Center for Fluorescence Spectroscopy, Department of Biochemistry and Molecular Biology, School of Medicine, University of Maryland School of Medicine, 725 Lombard Street, Baltimore, MD 21201, USA; E-Mails: jianzhang@umaryland.edu (J.Z.); jlakowicz@umaryland.edu (J.R.L.)

**Keywords:** zinc oxide nanostructures, single molecule spectroscopy, interfacial electron transfer, radiative decay rate

## Abstract

Single-molecule fluorescence spectroscopy has now been widely used to investigate complex dynamic processes which would normally be obscured in an ensemble-averaged measurement. In this report we studied photophysical behaviors of single fluorophores in proximity to zinc oxide nanostructures by single-molecule fluorescence spectroscopy and time-correlated single-photon counting (TCSPC). Single fluorophores on ZnO surfaces showed enhanced fluorescence brightness to various extents compared with those on glass; the single-molecule time trajectories also illustrated pronounced fluctuations of emission intensities, with time periods distributed from milliseconds to seconds. We attribute fluorescence fluctuations to the interfacial electron transfer (ET) events. The fluorescence fluctuation dynamics were found to be inhomogeneous from molecule to molecule and from time to time, showing significant static and dynamic disorders in the interfacial electron transfer reaction processes.

## 1. Introduction

Nanostructured metal oxide semiconductors, such as TiO_2_, SnO_2_, and ZnO, have attracted considerable attention as promising materials for optical devices in the past years mainly due to their attractive optical properties, for instance, wide direct band gap and large exciton binding energy. Sensitization of semiconductor nanostructures by organic dye molecules has also been studied for use in solar photovoltaic devices [1–4]. Semiconductors with a wide band gap are sensitized to visible light by surface-adsorbed dye molecules and charge transfer process occurs from dye molecules to the conduction band of the semiconductor [1,5–7]. Examples of ZnO applications include short wavelength light-emitters, field-emitters, luminescence, and UV lasers [8–12]. ZnO nanomaterials are stable in typical chemical environments and can be easily processed through numerous synthetic routes [8,12–16]. Therefore zinc oxide nanostructures show potential for advanced optical devices. Recently, zinc nanomaterials have been shown to increase fluorescence emission with emission wavelengths in the visible blue to red spectral range [17–19]. To date, noble metallic nanostructures have become well known to favorably modify the spectral properties of fluorophores and to improve some of their classical photophysical properties, such as increased intensity and enhanced photostability [20–23]. Fluorescence enhancement has been reported for fluorophores, quantum dots, and rare-earth complexes near nanostructure metals through coupling with surface plasmons in metallic nanostructures. Another exciting research direction for plasmonics research is photovoltaics. Design approaches based on plasmonics have been used for high-efficiency solar-cell design. Therefore, the fundamental importance and impending applications make the study of interactions between dye molecules and semiconductors a promising research area. Such interfacial interactions often involve significant heterogeneous dynamics and complex local nanoenvironments that are often difficult to analyze by traditional ensemble-averaged experiments. We herein report for the first single-molecule studies of photophysical behaviors of a near infrared (IR) cyanine dye (Cy5) on nanoscaled zinc oxide (ZnO) platforms. Single-molecule fluorescence spectroscopy has emerged as a powerful tool for imaging and is capable of probing complex systems and revealing a real-time landscape of dynamics [24–26]. As single fluorescent molecules are sensitive to their surrounding environments, profiling individual molecules and probing single molecules including fluorescence intensity time traces, spectra and fluorescence lifetimes can provide unique information about the chemical and physical characteristics of the local environment and reveal the static and dynamic heterogeneity. Single-molecule spectroscopy has been performed for sophisticated studies such as intersystem crossing [27,28], energy transfer [25,29], electron transfer [6,7,24] and spectral diffusion [30]. The interfacial electron transfer mechanisms of fluorophores on TiO_2_ semiconductor substrates are well documented [7,29,31–34]. Reports of fluorophore photophysical behaviors on ZnO are relatively sparse [35]. Our work demonstrates enhanced brightness observed from individual fluorophores adsorbed on ZnO nanostructure. Additionally, the single-molecule fluorescence trajectories show strong fluctuations and extended dark periods. Combining statistical analysis on fluorescence intensity and lifetime trajectories, we attribute these phenomena to heterogeneous reactivity of the interfacial electron transfer processes on ZnO nanostructures.

## 2. Results and Discussion

**Figure 1:**
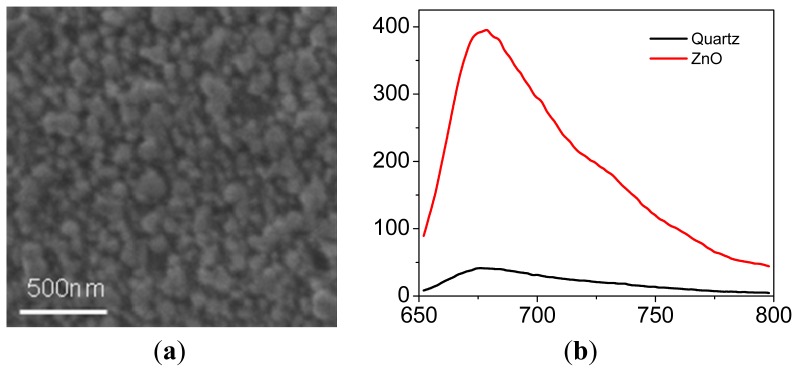
(**a**) A SEM image of ZnO; (**b**) Spectra of Cy5/PVA deposited on Quartz glass (black) and ZnO film (red), the Cy5 concentration for spin coating is around 0.1 μM. (**a**) (**b**)

SEM images reveal that ZnO film consists of hexagonal ZnO nanoclusters with a mean diameter of 100 nm (Figure 1a). Ensemble emission spectra of Cy5 on glass and ZnO surface with 630 nm excitation are shown in Figure 1b. It has been observed that the emission intensity increases approximately 10-fold as the dye molecules deposited on ZnO nanoparticle surface. In the presence of ZnO nanoparticles, the emission spectrum is slightly red-shifted; this can be contributed to the electronic coupling of the fluorophore and electronic states of the semiconductor [36].

**Figure 2:**
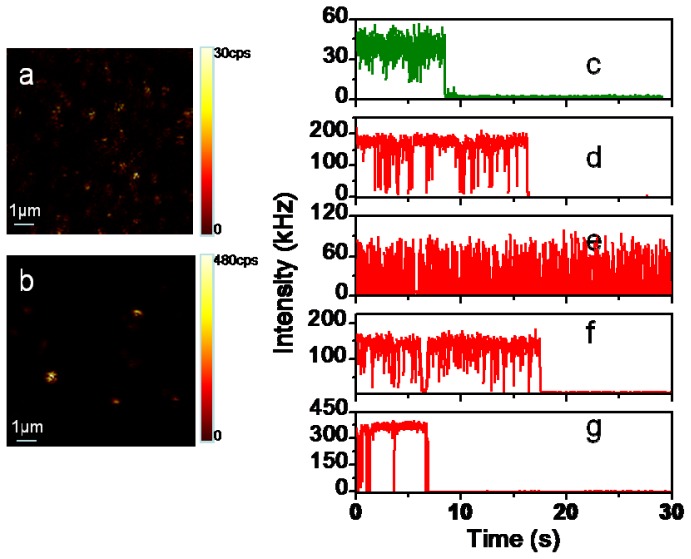
Fluorescence images for single Cy5 molecules on a cover glass (**a**) and a ZnO covered surface (**b**). Size: 10 μm × 10 μm. (**c**–**g**) typical single molecule traces of single Cy5 molecules on cover glass (**c**) and ZnO covered glass (**d**–**g**). The binning time is 1 ms. Single-molecule samples were obtained by spin-coating a polyvinyl alcohol (PVA) solution containing 1 nM Cy5 molecules on ZnO and on glass substrates, respectively.

**Figure 3:**
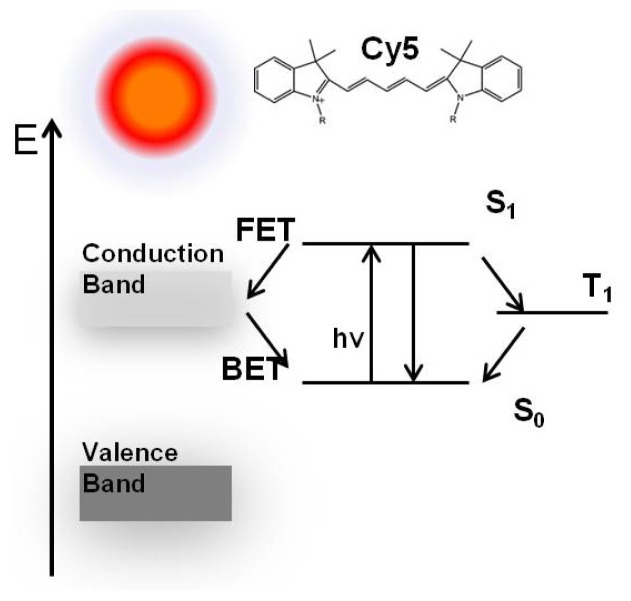
Schematic diagram of a model of photoinduced processes in a dye-deposited ZnO system. The ZnO energy levels are shown at the left, the relevant energy levels for the dye molecule are shown on the right. The optical transition between the ground electronic state (S_0_) and the excited electronic state (S_1_) is illustrated by the arrow labeled as *h*ν.

Single molecules were studied using a stage-scanning confocal microscope equipped with time-correlated single photon counting (TCSPC) module. Figure 2a,b show typical 10 μm × 10 μm images for single Cy5 molecules on a coverslip and a ZnO nanoparticle covered surface, respectively. The individual bright spots in the images are attributed to the single-molecule emission as evidenced by several criteria [37–39]. (1) The emission spot is about the same size order as the diffraction-limited size of the laser focus, which is about 300 nm in this confocal setup; (2) The signal brightness is comparable as expected for single-molecule emission considering the properties of the molecule, the excitation intensity, and the efficiency of optical filters; (3) The density of the spots increases proportional to the concentration of dye in the solution. Figure 2 shows characteristic fluorescence intensity time traces of single Cy5 molecules on bare glass (Figure 2c) and on ZnO substrate (Figure 2d–g), respectively. Typical single-molecule trajectories show constant fluorescence emission before photobleaching and the intensity drops to the background abruptly in a single step. Many traces of single fluorophores on ZnO exhibit pronounced fluctuations between bright and dark states over the course of their trajectories accompanied by a significant increase in emission rate as shown in Figure 2d–g. In contrast to the relatively continuous emission on glass surface in Figure 2c, increase in the number of “on/off” events was observed occasionally in the trajectories (Figure 2d–g) with “off” time ranging from sub-seconds to seconds. The fluorescence blinking observed from molecules on bare glass is contributed to temporary trapping of the excited states in long-lived triplet states of the dye molecules [27]. We believe that the single-molecule fluorescence intensity fluctuation is not due to triplet state blinking during the experiment. Under ambient conditions, the time scale of the triplet blinking is approximately shorter than 1 ms [27] which is clearly distinguished from the fluorescence intensity fluctuation time of subseconds to seconds as depicted in Figure 2d–g, the triplet blinking is thus not responsible for the observed long “off” times. Additionally, fluorescence counts depicted are integrated in 5 ms binning time, the fast fluorescence intensity fluctuations or blinking of the dye molecules (on sub-millisecond levels) are averaged out herein, as we observed a relatively steady fluorescence intensity level as illustrated in Figure 2c. It has been reported that excited organic fluorophores on a semiconductor coated surface show rapid reversible electron transfer and exhibit similar dramatic fluctuation behaviors [5–7,24,29]. The occurrences of the longer “off” time are clearly related to the proximity of ZnO surface and we assume that the stochastic fluctuation behaviors such as those in Figure 2d–g are the results of electron transfer (ET) processes to the ZnO nanostructures: electron injection from the fluorophore into the conduction band of the semiconductor leads to the loss of fluorescence, and charge recombination leads to the return of fluorescence [5,24]. A similar model of the photophysical processes may be represented by Figure 3. The forward electron transfer (FET) kinetics in various dye-semiconductor systems have been studied with rates in the femtoseconds to several hundred picoseconds range, the injected electron is then localized on the ZnO semiconductor surfaces followed by a backward electron transfer (BET) process. Electron injection from the fluorophore into the conduction band of the ZnO leads to the loss of fluorescence, and charge recombination leads to the return of fluorescence. The long “off” periods in the fluorescence trajectory is herein the result of electron transfer process quenching the fluorescence, and the “on” state is due to natural fluorescence emission cycles.

**Figure 4:**
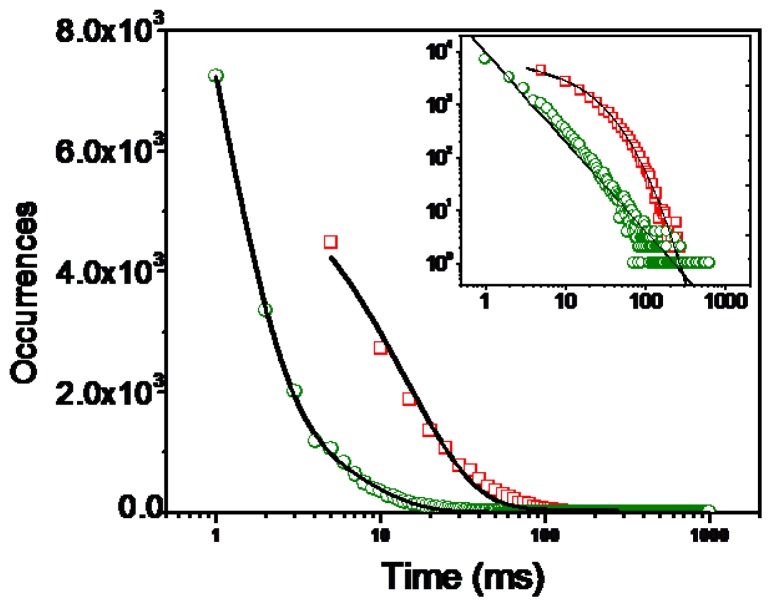
Histograms and exponential fits of the off time durations (green open circle: molecules on glass; red open square: molecules on ZnO surfaces). The solid line over open circles show a single exponential fit for single Cy5 molecules dispersed on glass: *τ*_off_ = 1.73 ± 0.02 ms. The inserts show log−log scale off time distributions.

The duration of the “off” states extends over a broad range from subseconds to seconds. To analyze the single molecule interfacial electron transfer dynamics that is associated with the fluctuation of the single-molecule fluorescence trajectories, we have performed statistical analysis on the stochastic durations of the dark states in which the high ET activity takes place. A method similar to the previously used to analyze triplet blinking was used: a threshold was determined at a level of 2-fold standard deviation with respect to the mean background during the off time histogram analysis to distinguish between the states as described elsewhere [38,39]. A combined off-time distribution from more than 20 molecules is used herein. The time duration of the dark states are then obtained. The histogram of the off time on bare glass is fitted with a single-exponential curve and yields an average off time (*τ*_off_) of 1.73 ± 0.02 ms. The log-log distribution of Cy5 molecules on bare glass substrate can be fitted to a straight line (the insert in Figure 4). We can describe this off time distribution using a power law function *p*(*τ*_off_) = *p^0^τ*_off_^–^*^α^*^_0ff_^, with *α*_0ff_ being the power law exponent. The single molecule power exponent for the off-time statistics is sensitive to the molecule environment and/or the molecule structure. It is obvious from the linear log-log plot that the observed off-times on glass coverslips covers a range of times and can be described using a power law distribution. The *α*_0ff_ value of 1.50 of Cy5 molecules in the absence of ZnO is in agreement with other organic fluorophores obtained on glass substrates [40], which suggests that the molecular system is well behaved and the blinking is due to a single process. It is interesting that for single molecules on ZnO surfaces, multi-exponential behavior is observed in the dark time distribution shown in Figure 4. This multi-exponential distribution suggests that the interfacial ET involves complex processes that cannot be defined by a static rate constant and the rate changes from time to time, and lead to a dramatic change in “off” times. The electron transfer reactivity can be reflected by the fluorescence emission fluctuation. As electron transfer reactivity fluctuates, fluorescence shows dramatic fluctuations and gives striking bright and dark states. The intermittency and fluctuation of the single-molecule fluorescence are then attributed to the differences of the reactivity of interfacial electron transfer at the single molecule/ZnO nanoparticle interfaces [35,41,42]. The “off” periods in the fluorescence trajectories are accounted for by an electron transfer process with high activity quenching the fluorescence and the “on” periods are owing to low activity in ET process.

**Figure 5:**
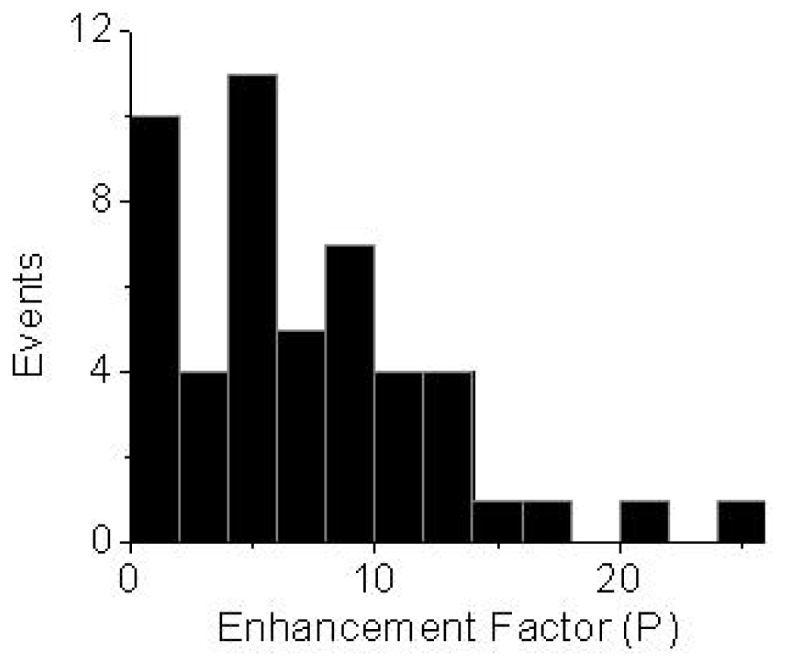
Enhancement factor distribution of single Cy5 molecules on ZnO surfaces. The histogram was constructed from more than 50 molecules.

The fluorescence emission rates of single probes on glass coverslips covered with ZnO were enhanced or remained the same on glass. A large distribution of single molecules shows as much as more than 10-fold increase in brightness when deposited on ZnO (Figure 5), which is in good agreement with ensemble experiment results. Zinc nanostructures have been reported to enhance emission of fluorophores [17–19] and such effects were ascribed to the enhanced absorption rate [17] in close proximity to zinc films and the possible changes in photonic mode density or reduction in self-quenching of fluorophores near ZnO nanostructures [18,19]. The exact mechanism governing the enhanced fluorescence on ZnO is still under investigating. In our approach, we assume the incident light is trapped by scattering so the effective intensity is increased, leading to enhanced fluorescence emission. The SEM image of ZnO film shows distinct features on the nanometer scale, making a heterogeneous environment available on the surface, as a result variation in brightness of the fluorophores was observed.

**Figure 6:**
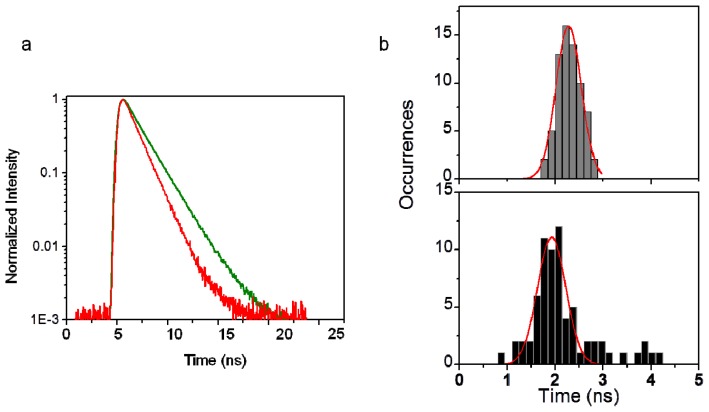
(**a**) The typical decay curves on glass (green line) and on ZnO surface (red line); (**b**) Lifetime histograms of single Cy5 molecules on glass (top, gray) and ZnO covered glass (bottom, dark). Red line: Gaussian fits. The histograms are constructed from more than 50 molecules on glass and ZnO, respectively.

The time-correlated single photon counting (TCSPC) lifetime measurements could be used to monitor the change in radiative rates. An increase in radiative rates could result in a significant reduction in the intrinsic lifetime, improvement in photostability, and suppression of blinking [22,39]. By using pulsed excitation and measuring the delay between the arrival time of the excitation pulse and the detection of the emitted photon, it is possible to reconstruct a decay curve for the excited state of the fluorophore. The measured total fluorescence decay rate is the sum of two independent contributions, the radiative decay rate and the nonradiative decay rate [43]. The decay time is often mono-exponential and given by

(1) $$\tau ={\left(\Gamma +k_{nr}\right)}^{-1}$$

where *Γ* and *k*_nr_ are the radiative and non-radiative decay rate, respectively. Suppose a fluorophore displays a low quantum yield, which implied *k*_nr_ >> *Γ*, as the value of *Γ* increases, the lifetime decreases. The changes in *k*_nr_ are typically due to changes in an emitter’s environment, quenching or resonance energy transfer (RET). The radiative decay rate *Γ* is constant and the changes are primarily due to changes in dielectric constant. Many fluorescence decays of single Cy5 molecules on glass can be fitted to a single component exponential function and the measured lifetimes are equal to the intrinsic lifetime. Figure 6 shows examples of time-resolved decay curves derived from trajectories illustrated in Figure 2c,e, respectively. The fluorescence intensity decays were analyzed in terms of the multi-exponential model as the sum of individual single exponential decays [44]:

(2) $$\text{I}(t)=\sum_{i=1}^{n}\alpha_{i}e^{\left(-\frac{t}{\tau_{i}}\right)}$$

where *τ_i_* is the decay time and *A_i_* is the amplitude. The fractional contribution of the component to the intensity is described by:

(3) $$f_{i}=\frac{\alpha_{i}\tau_{i}}{\Sigma_{i}\alpha_{i}\tau_{i}}$$

The averaged lifetime is expressed by:

(4) $$\tau_{avg}=\sum_{i}f_{i}\tau_{i}$$

The values of *α_i_* and *τ_i_* were determined using the PicoQuant Symphotime software with nonlinear least square fittings. The curves in Figure 6a decay mono-exponentially with a time constant of 2.44 ns for a single fluorophore dispersed on the bare glass. A rather faster decay process of Cy5 on ZnO derived from Figure 2g was observed with a bi-exponential fit with an averaged time constant of 1.53 ns, giving a long component of 2.82 ns, 25% amplitude, with a short component of 1.11 ns, 75% amplitude. The decays of single molecules on ZnO can be better described by two-exponential fittings. The results of the fits derived from time trajectories in Figure 2d–g are shown in Table 1. The small change in lifetimes on ZnO suggests that the increased fluorescence is due to the increased excitation, rather than a large change in the radiative rate. A short component ranging from 800 ps to 1.6 ns and a long component of approximately 2–6 ns were normally found for the molecules on ZnO surface. However, there is no correlation between the fluctuation in lifetime and intensity. To quantify the fluorescence lifetime distribution of single molecules, we constructed the averaged lifetime histograms of more than 60 single molecules on bare glass and on ZnO, respectively, as shown in Figure 6b with each distribution fitting to a Gaussian function. The mean of the lifetime distribution is 2.28 ± 0.01 ns on glass. The measured lifetimes on ZnO surfaces presents a trend of broadening distribution and yields a mean value of 1.93 ± 0.02 ns. The peak position shifted to shorter lifetimes and width is much broader compared to those on glass. For a fluorophore at an interface, its radiative lifetime depends on the refractive index of the media and the orientation of the molecule relative to the interface normal. A distribution of single molecule lifetimes on glass is attributed to disparity of orientation [27]. Although the higher index refraction of the ZnO (*n* = 2.00) could result in a slightly larger radiative rate compared to that on glass (*n* = 1.52), we observed diverse intensity decay components of single molecules on ZnO surfaces, the natural radiative lifetime is unlikely to vary so dramatically under these single-molecule experimental conditions. There should be some competition between direct electron transfer from the excited state of the dye molecule to the ZnO conduction band and other radiative and non-radiative excited state relaxation pathways, therefore the variations in lifetime components imply additional non-radiative decay pathway arising from electron transfer, and the fluorescence lifetime is thus slightly modified. The relatively shorter averaged fluorescence lifetime of single molecules on ZnO can be attributed to the presence of electron transfer activity. The observation of broadened lifetime distribution reveals the heterogeneity of site-specific molecule interactions. When the electron transfer process activity is low, the radiative emission plays a key role and the observed fluorescence intensity is high, giving the bright or even enhanced signal. If photoinduced electron transfer occurs, the ET process quenches the radiative emission and becomes an additional nonradiative decay pathway for the excited state, the detected fluorescence signal is low and the fluorescence lifetime is altered. In these trajectories, the variations in lifetime suggest a fluctuation in the non-radiative decay rate. It is interesting to note that long component of intensity decays of a few observable molecules are around 4–5 ns, which is much longer than the fluorophore’s averaged lifetime. These molecules may inject electrons at low rates.

## 3. Experimental Section

### 3.1. ZnO Film Preparation

The synthesis of ZnO film was based on a published report [13]. Glass coverslips were cleaned with chromic acid followed by ultrasonic bath cleaning. Zn(NO_3_)_2_ and NaOH were used to prepare homogeneous solution of sodium zincate. The deposition of thin films was carried out by dipping the glass coverslips in sodium zincate (0.04 M) solution followed by dipping in hot water maintained at 90 °C. Each time the substrate was kept in the bath and hot water for 30 s, respectively. To obtain smooth films, the process was repeated 20 times. Only one side of glass was coated with zinc oxide film. The surface morphology of ZnO nanostructures was observed using Quanta 200 (FEI) scanning electron microscopy.

### 3.2. Bulk Fluorescence Measurements

Emission spectra from Cy5 dispersed on glass and ZnO substrates were collected in front-face geometry on a SLM 8000 spectrofluorometer with 635 nm excitation from a xenon lamp. Cy5 molecules were spin-cast onto the substrates from an aqueous solution containing 0.5% polyvinyl alcohol (PVA). The concentration of the Cy5 sample in the solution was 100 nM.

### 3.3. Single-Molecule Fluorescence Spectroscopy and Imaging

Single-molecule fluorescence images and intensity time traces were recorded with a time-resolved confocal microscope (MicroTime 200, PicoQuant). A single mode pulsed laser diode (635 nm, 100 ps, 40 MHz) (PDL800, PicoQuant) was used as the excitation light. The collimated laser beam was spectrally filtered by an excitation filter (D637/10, Chroma) before directing into an inverted microscope (Olympus, IX 71). An oil immersion objective (Olympus, 100×, 1.3 NA) was used both for focusing laser light onto the sample and collecting fluorescence emission from the sample. The fluorescence that passed a dichroic mirror (Q655LP, Chroma) was focused onto a 75 μm pinhole for spatial filtering to reject out-of-focus signals. To further isolate single-molecule emission and reduce background, the desired spectral detection range was selected by placing a bandpass filter (HQ685/70, Chroma) in front of the single photon avalanche diode (SPAD). Images were recorded by raster scanning (in a bidirectional fashion) the sample over the focused spot of the incident laser with a pixel integration of 0.6 ms. The excitation power into the microscope was maintained less than 1 μW. Time-dependent fluorescence data were collected with a dwell time of 50 ms. The fluorescence lifetime of single molecules was measured by time-correlated single photon counting (TCSPC) with the TimeHarp 200 PCI-board (PicoQuant). The data was stored in the time-tagged-time-resolved (TTTR) mode, which allows recording every detected photon with its individual timing information. Fluorescence decay curves were fitted to biexponential functions. In combination with a pulsed diode laser, total Instrument Response Function (IRF) widths of about 400 ps FWHM can be obtained, which permits the recording of sub-nanosecond fluorescence lifetimes, extendable to less than 100 ps with reconvolution. All measurements were performed in a dark compartment at room temperature. Samples for single-molecule measurement were prepared as described above, the concentration of Cy5 in 0.5% PVA solution was adjusted to the nanomolar level to give an appropriate surface density for the observation of single molecules.

## 4. Conclusion

In summary, individual fluorophore molecules were deposited on ZnO films as probes of interfacial electron transfer dynamics, which should help to understand the intrinsic properties of electron transfer at interface between organic molecules and transparent semiconductor materials. We observed enhanced fluorescence of single fluorophores dispersed on ZnO surfaces accompanied by dramatic intensity intermittency and fluctuations. Such intense blinks are rarely observed when ZnO nanostructures are not present and could be attributed to the interfacial electron transfer. The static heterogeneity originates from different interfacial reactivity fluctuations from molecule to molecule, presumably due to the variable roughness of ZnO surface, leading to fluorescence fluctuations, whereas dynamic heterogeneity is associated with the electron transfer reactivity fluctuations from time to time stochastically for the same individual molecules, resulting in different time spans of the on/off periods. If a photoinduced electron transfer occurs, then electron transfer becomes an additional nonradiative decay pathway for the excited state, the fluorescence lifetime is modified. Since the radiative lifetime is not expected to vary so dramatically, the change is mainly attributed to electron transfer between the photoexcited fluorophore and the conduction band of the semiconductors. However, the ET activity is intermittent in nature and dominates the fluorescence process only by a fraction of time. Each on/off period likely includes occurrences of multiple electron transfer events. The time-resolved fluorescence studies also confirm that the observed enhanced fluorescence from ZnO nanostructures films is mostly due to the scattering effect. Interfacial electron transfer processes play a key role in many chemical and biological processes. The heterogeneous photophysical behaviors of single molecules and the lifetime distribution clearly demonstrate an ET dynamics, which is revealed in the single-molecule experiments better than in the ensemble experiments. Though investigation of electron-transfer processes at the single molecule level is still a relatively novel practice, it has shown to be promising. Since there is a clearly increased interest in the study of single molecule electronic processes from the molecular electronics field, we believe the studies will make important contributions in areas such as in solar cells and biological photosystems, where photoinduced electron transfer processes are of significance.

## Figures and Tables

**Table 1:** Exponential fitting paramters of intensity decay curves derived from time trajectories shown in Figure 2.

Trace	*α*_1_	*τ*_1_ (ns)	*α*_2_	*τ*_1_(ns)	*τ*_avg_(ns)
d	0.086	1.38	0.914	2.49	2.4
e	0.888	1.34	0.112	1.49	1.35
f	0.076	1.12	0.924	1.92	1.86
g	0.753	1.11	0.247	2.82	1.53
